# Complete Genome Sequence of Bifidobacterium longum subsp. infantis Strain CECT 7210, a Probiotic Strain Active against Rotavirus Infections

**DOI:** 10.1128/genomeA.00105-15

**Published:** 2015-04-02

**Authors:** Empar Chenoll, Montserrat Rivero, Francisco M. Codoñer, Juan F. Martinez-Blanch, Daniel Ramón, Salvador Genovés, Jose Antonio Moreno Muñoz

**Affiliations:** a Department of Food Biotechnology, Biópolis SL, Paterna, Valencia, Spain; b Ordesa SL, Barcelona, Spain; c Lifesequencing SL, Paterna, Valencia, Spain

## Abstract

Bifidobacterium longum subsp. infantis CECT 7210 is a probiotic strain able to inhibit rotavirus *in vitro* and protect against viral infection in both cell cultures and mice. Here, we report its complete genome sequence, as deciphered by PacBio single-molecule real-time (SMRT) technology. An analysis of the sequence may provide insights into its functional activity.

## GENOME ANNOUNCEMENT

It is widely accepted that probiotics boost the immune system and exert an *in vivo* antimicrobial effect in humans (1). In this case, the strain Bifidobacterium longum subsp. infantis CECT 7210, isolated from fecal samples of breastfed infants, proved to exert a direct effect on rotavirus infection *in vitro* in MA-104 and HT-29 cell lines and to provide *in vivo* preliminary protection against the rotavirus strain McN infection in a mouse model (2). Furthermore, an increase in IgA antibody levels after oral administration of the strain was observed (2). The aforementioned results, together with its resistance to gastrointestinal juices, bile salts, NaCl, and low pH, as well as adhesion to intestinal mucus and sensitivity to antibiotics, demonstrated that strain CECT 7210 can be considered a probiotic able to inhibit rotavirus infection (2). Here, we present the complete genome sequence of this probiotic strain.

In order to carry out the complete genome sequencing of the strain B. longum subsp. infantis CECT 7210, massive sequencing technology was implemented at the PacBio platform (Pacific Biosciences, Menlo Park, CA). A 10-kb library was constructed with purified DNA, and six single-molecule real-time (SMRT) cells were sequenced using XL-C2 chemistry and a data collection time of 120 min. The sequencing run provided a total amount of 224,000 sequences with an accuracy of Q20. The obtained sequences were filtered by quality, and a total of 181,032 sequences were obtained, with a mean read length of 4,301 nucleotides (nt) of quality Q20. The total data output was 778.6 Mb. A *de novo* assembly employed the parameters by default with the Hierarchical Genome Assembly Process approach (HGAP). Finally, 8,140 reads of around 8 kb were obtained, providing a unique contig of 2,467,698 base pairs and coverage of 251×. After clipping an overlapped region at the end of the contig, the genome was circularized with an estimated size of 2,455,085 nt. No plasmids were detected.

The genome contains 2,171 elements, of which 2,072 are open reading frames (ORF) (1,838 canonical and 234 noncanonical). Of the 2,171 elements, 69 are structural RNAs (sRNAs) (12 rRNAs and 57 tRNAs). To search in depth for similarities between the CECT 7210 strain and other B. longum strains, the B. longum CECT 7210 genome was compared with other B. longum reference genomes (i.e., B. longum 157F). In the case of B. longum subsp. infantis 157F (3) (genome, GenBank accession no. NC_015052), we found that the CECT 7210 genome contains 340 elements missing in the probiotic strain 157F. In this respect, an analysis of the complete genome of B. longum CECT 7210 may help us understand the mechanisms involved in its protective effect against rotavirus.

### Nucleotide sequence accession number.

The results of the whole-genome project have been deposited at DDBJ/EMBL/GenBank under the accession no. LN824140. The version described here is the first version.
